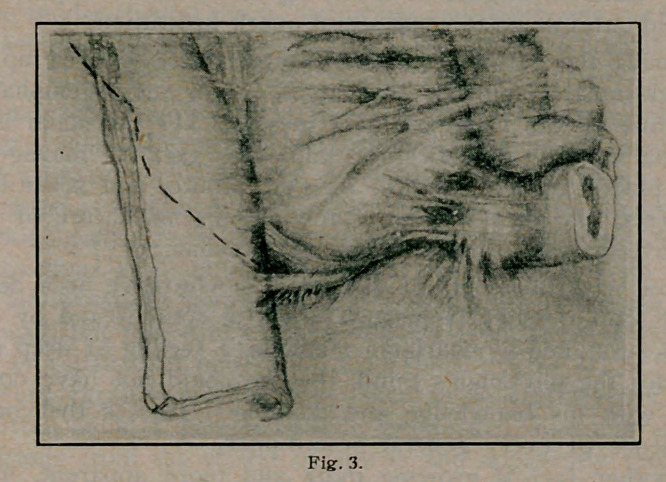# Abnormalities of the Appendix Due to Disease

**Published:** 1912-12

**Authors:** Joseph S. Lewis

**Affiliations:** Buffalo N. Y.; 471 Viriginia Street


### BUFFALO MEDICAL JOURNAL

VOL. LXVIII DECEMBER, 1912 No. 5

### ORIGINAL ARTICLES

### Abnormalities of the Appendix Due to Disease

JOSEPH S. LEWIS.
Buffalo N. Y.

ABNORMAL and curious conditions of the vermiform appendix are of so frequent occurence that only the most striking and eccentric usually find their way into the literature.

The occurence of double appendix is so rare as to cast doubt on the few cases reported. Absent appendix can be accounted for on morphologic grounds as well as through the accidents of previous disease. The writer once assisted at an operation where, even after enlargement of the incision and painstaking search, by a careful surgeon, of an otherwise normal caecum, no sign of any appendix was found save a small conical protrusion of the gut where the appendix should have been.

Some two years ago in a notorious book on the troubles and failures of the medical profession there was cited a case since often repeated with gusto by laymen. A surgeon having, as he thought removed the appendix of a certain patient, was chagrined on seeing a colleague later remove an appendix from the same patient. That this story need not reflect on the ability of surgeon No. 1, the following instances will tend to prove. These are not intended as case reports but conditions observed by the writer in his own experience and in his hospital service as interne.

(Fig 1.) This patient had two years previously an appendix abscess which was incised and drained. At the second operation the distal end of the appendix was found attached like a captive dirigible baloon by a thin mesappendix to a short proximal stump under adhesions. This stump need have been only a trifle longer to have made possible a second appendicitis. The writer marvels, that the apparently normal, though somewhat atrophic mucosa within the free portion, had not distended it with secretion as has been shown to occur in an excluded closed loop of gut.

(Fig. 2.) This represents a type in which the proximal end of the appendix lies retrocaecal. The writer has seen a second rate surgeon leave behind the retrocaecal one-third of such an ap-

Fig. 1

pendix through use of the absolute method of simple ligation of the appendix leaving uncovered the cauterized stump. Investigation would have disclosed the base.

(Fig 3.) This patient was operated on in extremis with peritonitis, by a most capable surgeon. At a secondary operation

Fig. 2

by the same man for adhesions, remnants of an appendix were removed from dense adhesions. At a third operation for ileocaecal obstruction the writer found one and one-fourth inches of appendix distally adherent to the drainage scar in the abdominal wall.

These experiences confirm the value of burial of the appendix stump in the caecal wall and afford an explanation for some cases of apparent but not actual removal of the appendix.

471 Viriginia Street.

## Figures and Tables

**Fig. 1 f1:**
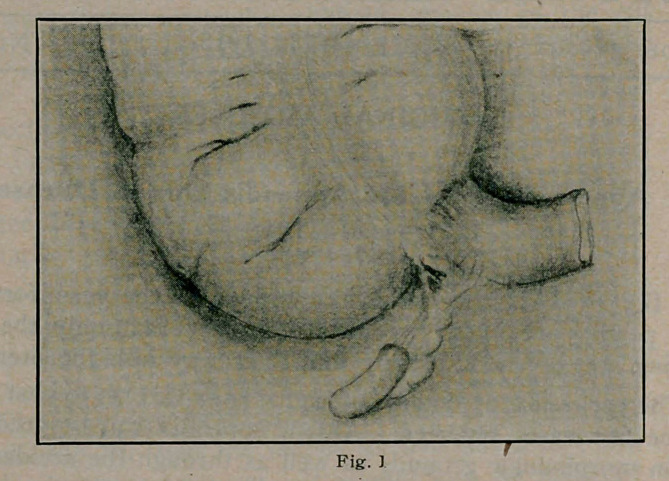


**Fig. 2 f2:**
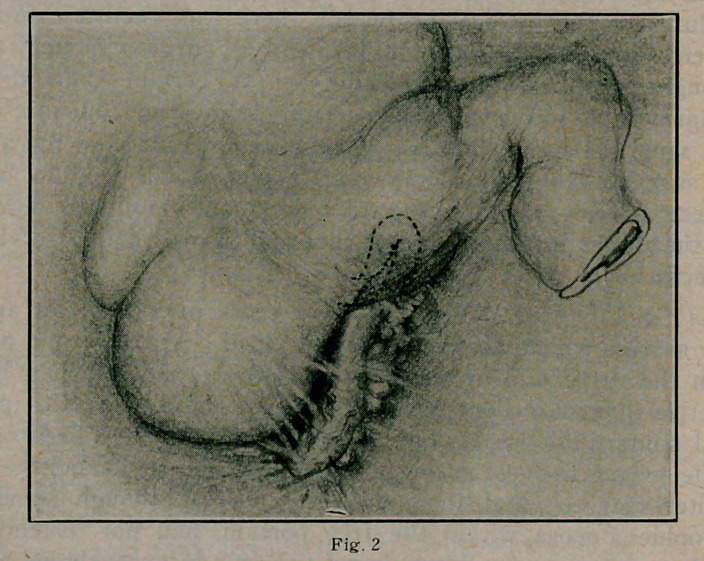


**Fig. 3. f3:**